# Aqueous extract of *Arctium lappa L.* root (burdock) enhances chondrogenesis in human bone marrow-derived mesenchymal stem cells

**DOI:** 10.1186/s12906-020-03158-1

**Published:** 2020-11-23

**Authors:** King-Chuen Wu, Hung-Kai Weng, Yun-Shang Hsu, Pin-Jia Huang, Yang-Kao Wang

**Affiliations:** 1 Department of Nursing, Chang Gung University of Science and Technology, Chia-Yi County, Taiwan; 2 Department of Anesthesiology, Chang Gung Memorial Hospital, Chiayi County, Taiwan; 3 Department of Orthopaedics, National Cheng Kung University Hospital, College of Medicine, National Cheng Kung University, Tainan City, Taiwan; 4 Institute of Basic Medical Sciences, College of Medicine, National Cheng Kung University, Tainan City, Taiwan; 5 Department of Cell Biology and Anatomy, College of Medicine, National Cheng Kung University, Tainan City, Taiwan

**Keywords:** Aqueous extract of *Arctium lappa L.* root, Mesenchymal stem cells, Chondrogenic differentiation

## Abstract

**Background:**

*Arctium lappa L.* root (burdock root) has long been recommended for the treatment of different diseases in traditional Chinese medicine. Burdock root possesses anti-oxidative, anti-inflammatory, anti-cancer, and anti-microbial activities. The aim of the study was to elucidate whether aqueous extract of burdock root regulates mesenchymal stem cell proliferation and differentiation.

**Methods:**

Human bone marrow-derived mesenchymal stem cells in 2D high density culture and in 3D micromass pellets were treated with chondrogenic induction medium and chondral basal medium in the absence or presence of aqueous extract of burdock root. The chondrogenic differentiation was accessed by staining glucosaminoglycans, immunostaining SOX9 and type II collagen and immuonblotting of SOX9, aggrecan and type II collagen.

**Results:**

Treatment of aqueous extract of burdock root increased the cell proliferation of hMSCs. It did not have significant effect on osteogenic and adipogenic differentiation, but significantly enhanced chondrogenic induction medium-induced chondrogenesis. The increment was dose dependent, as examined by staining glucosaminoglycans, SOX9, and type II collagen and immunobloting of SOX9, aggrecan and type II collagen in 2D and 3D cultures. In the presence of supplemental materials, burdock root aqueous extract showed equivalent chondrogenic induction capability to that of TGF-β.

**Conclusions:**

The results demonstrate that aqueous extract of *Arctium lappa L.* root promotes chondrogenic medium-induced chondrogenic differentiation. The aqueous extract of burdock root can even be used alone to stimulate chondrogenic differentiation. The study suggests that the aqueous extract of burdock root can be used as an alternative strategy for treatment purposes.

**Supplementary Information:**

**Supplementary information** accompanies this paper at 10.1186/s12906-020-03158-1.

## Background

Osteoarthritis (OA) is a chronic disease, which causes the degeneration of joint cartilage and the underlying bone. In severe condition, the cartilage breaks down, and the joint space becomes narrow, resulting in the exposure of periarticular bone and soft tissues. This can cause pain, swelling, misshapenness, and disability within the joint, and also irritation in the surrounding tissues [1]. OA is the most common form of arthritis, affecting tens of millions of people worldwide. In the United State of America, according to the National Health Interview Survey, 14 million people are suffering from symptomatic knee OA [2]. Since the frequency of OA increases with age, the number of OA cases is expected to increase in the next decade [3].

Current treatment strategies for OA include: managing the pain, improving the mobility of the joint, minimizing disability, and restoring joint function. The treatment types for OA include the use of medications and non-pharmacological therapies. The current pharmacological options are limited to painkillers and anti-inflammatory drugs. Such treatments may be ineffective, or even lead to severe adverse effects, such as stomach problems, high blood pressure, etc., in patients [4]. Study reported the application of Chinese herbal medicine alone (CHM) or in combination with routine protocols for the treatment of OA [5]. Though CHM, together with routine protocols possesses considerable therapeutic effect in the treatment of knee OA without too much side effects, more studies are needed to clarify the efficacy and safety of these treatments.

Recently, stem cell-based therapies directed the attention towards trials for the treatment of OA. Among different stem cells, human mesenchymal stem cells (hMSCs) from adult origins, mostly bone marrow and adipose tissues, are capable of differentiating into tissues including bone, cartilage, and fat [6]. These MSCs are easy to isolate and have good proliferation potential. MSCs maintain their differentiation capability at early passages, and elicit low immunological rejection due to their low expression of major histocompatible antigens [6, 7]. Previous reports demonstrated that human MSCs (hMSCs) can be differentiated into chondrogenic lineage by transforming growth factor β (TGFβ) [8, 9], fibroblast growth factors (FGFs) [10, 11], bone morphogenetic proteins (BMPs) [12–14], and insulin-like growth factor 1 [15, 16] in the presence of supplemental factors such as glucocorticoid and ascorbic acid [6, 17].

Successful chondrogenic differentiation can be detected by chondrogenic markers such as SRY-box transcription factor 9 (SOX9), type II collagen (collagen II), cartilage oligomeric matrix protein, and aggrecan [12, 18, 19]. Additionally, chondrogenic specific glycosaminoglycans (GAGs) can be observed via alcian blue staining [6, 17]. The above results suggest that hMSCs are a promising cell source for cartilage tissue regeneration. However, the application of the above cytokines/growth factors in the induction of chondrogenic differentiation could also result in adverse effects. For example, hypoglycemia, seizures, jaw pain and other side effects have been reported after administration of recombinant human IGF-1, FGF-2, BMP-2 and TGF-β [20–25].

*Arctium lappa L.*, also known as burdock, is a popular edible perennial plant used in traditional Chinese medicine (TCM) in Eastern Asia and in other countries for therapeutic purposes for hundreds of years. The burdock root has a unique flavor and a rich texture. It has been recommended as nutritive food in many countries. The burdock root contains lots of dietary fibers which promote intestinal peristalsis and defecation [26]. The major part of burdock for treatment purposes is the root, whereas other parts such as leaves [27, 28], fruits, and seeds [29, 30] are also used for different purposes. Studies demonstrated that burdock root extracts with different formulas possess anti-inflammatory [31–34], anti-mutagenic [35, 36], anti-tumorigenic [37], anti-oxidant [31, 38, 39], and anti-bacterial and anti-viral activities [40–42]. These studies suggest that burdock root extract is effective in the treatment of diseases [33, 43]. The burdock root extract contains active ingredients with different therapeutic effects against diseases [44, 45]. Though numerous reports demonstrated possible clinical uses of burdock root extract due to its above mentioned effects, its ability of inducing proliferation and differentiation of mesenchymal stem cells was not subject to research. Previous study reported to perform drinking burdock root tea for patients with knee OA and showed promising therapeutic results in anti-inflammation [33]. Furthermore, in TCM, the herbs were usually cooked in water and then gave to patients for disease treatment. These results suggest these aqueous solutions may contain effective ingredients which are beneficial for disease treatment. In this study, we investigate the role of the aqueous extract of burdock root in the regulation of proliferation and differentiation of human bone marrow-derived mesenchymal stem cells.

## Methods

### Experimental system

Primary human bone marrow-derived mesenchymal stem cells (hMSCs) were purchased from Lonza (Walksville, MD, USA). These cells were maintained in growth medium (GM) containing Dulbecco’s Modified Eagles Medium (low glucose) (DMEM, HiMedia, Mumbai, India) plus 10% fetal bovine serum (FBS) with selected lot (ThermoFisher Scientific, Waltham, MA, USA), 100 units/ml penicillin and 100 μg/ml streptomycin (Caisson Labs, Smithfield, UT, USA) at 37 °C with humidified 5% CO_2_ atmosphere according to established protocols [6, 46, 47]. The medium was changed twice a week. Only early passages (mostly P4-P5 and some P6) were used for our experiments.

### Induction of differentiation

For osteogenic differentiation, hMSCs were seeded at low density (3000 cells/cm^2^) and treated with osteogenic induction medium (OIM, GM plus 0.1 μM dexamethasone, 50 μM L-ascorbic acid-2-phosphate, 10 mM β-glycerophosphate. All purchased from Merck, Darmstadt, Germany). The medium was changed twice a week. After 2 weeks of treatment, the cells were fixed and stained for alkaline phosphatase activity (Merck). To induce adipogenic differentiation, hMSCs were seeded at high density (20,000 cells/cm^2^) and treated with adipogenic differentiation medium (AIM, GM, plus 1 μM dexamethasone, 0.1 μM indomethacin, 250 μM 3-isobutyl-1-methylxantine, and 10 μg/ml human recombinant insulin. All purchased from Merck). After 2 weeks of treatment, the cells were fixed and stained for lipid droplet using Oil red O [6, 46]. For both osteogenic and adipogenic induction, GM treatment was performed as a control. To induce chondrogenic differentiation, hMSCs were seeded at high density (20,000 cells/cm^2^) and treated with chondrogenic induction medium (CIM), containing DMEM low glucose, 0.1 μM dexamethasone, 50 μM L-ascorbic acid-2-phosphate, 1x Insulin-Transferrin-Selenium, 40 μg/ml L-proline, 1x L-glutamine (all purchased from Merck) and 5 ng/ml recombinant human Transforming growth factor β3 (Peprotech, Cranbury, NJ, USA) [6, 19, 47]. For 3D micromass culture, 3 × 10^5^ cells were centrifuged to make a pellet [6, 19] and CIM with and without aqueous burdock extract was added. The medium was changed twice a week. For high density culture, the cells were seeded overnight at a density of 2 × 10^4^/cm^2^ [47]. The next day, the medium was switched to CIM with and without different dosages of aqueous burdock root extract. For chondrogenic induction, cells treated with DMEM containing 1x Insulin-Transferrin-Selenium was performed as control. The medium containing chondrogenic supplements without TGF-β is termed as chon basal.

### The aqueous extract of burdock roots

We purchased the dried powder of aqueous extract of burdock roots from BioBest Co., Ltd. Taichung, Taiwan. The BD powder was dissolved in DMEM to make concentration of 100 mg/ml as stock and aliquoted and stored at − 20 °C. The remaining powder was stored in desiccator at room temperature.

### Measurement of cell proliferation using MTT assay

hMSCs at 3 × 10^4^/cm^2^ were seeded in a 96-well plate, and the next day, the medium was switched to CIM with and without burdock extract. After treatment, 3-(4, 5 Dimethylthiazol-2-yl)-2,5-Diphenyltetrazolium Bromide (MTT) reagent (Stock: 5 mg/ml, 20 μl/well, Merck) was added to each sample and incubated for 4 h. After removal of MTT solution, 100 μL of DMSO was added to each well, mixed, and incubated at 37 °C for another 30 min. The absorbance of each sample was read at 570 nm by an ELISA plate reader (μQuant Universal Microplate Reader, Bio-Tek, Winooski, VT, USA).

### Staining and quantification of glysosaminoglycans (GAGs) by alcian blue

The pellets and the high density culture of hMSCs were rinsed in phosphate-buffered saline (PBS) and fixed in 4% paraformaldehyde for 10 min. The pellets were embedded in Optimal cutting temperature (OCT) compound (Fisher Scientific, Pittsburg, PA, USA) and cryosectioned (LEICA CM 1950 Cryostat, Leica Biosystems, Buffalo Grove, IL, USA) into 10 μm thick slices. The cells and the cryosectioned samples were stained with 1% alcian blue (Merck). Then the samples were rinsed with PBS until the blue color disappeared from the negative control cells. The alcian blue-stained GAGs were quantified as per Wood et al. [48] with modification. Briefly, the alcian blue-stained GAGs were dissolved in 6 mol/L guanidine hydrochloride overnight at 4 °C. The absorbance of the dissolved alcian blue-stained GAGs was quantified at OD 620 nm using a spectrophotometer (μQuant Universal Microplate Reader).

### Immunostaining and immunofluorescence

The pellets of the hMSCs were rinsed by PBS, fixed in 4% paraformaldehyde for 10 min, and cryosectioned to 10 μm thick sections. The high density cultured hMSCs was rinsed with PBS and fixed with 4% paraformaldehyde for 10 min. Both the high density culture and the cryosectioned samples were rinsed with PBS, soaked in SuperBlock blocking buffer (Thermo) for 1 h at room temperature, then incubated with primary antibodies (SOX9, 1:200, cat. No#GTX109661; collagen II, cat. No# GTX20300; both antibodies were purchased from GeneTex, Irvin, CA, USA) overnight at 4 °C. Then the samples were incubated with secondary antibody conjugated with fluorophors (1:400, Alexa Fluor 488-conjugated goat anti-rabbit IgG, code No. 111–545-003, to SOX9; 1:400, Rhodamine-conjugated goat anti-rabbit IgG, code No. 111–025-003 to collagen II. Both antibodies were purchased from Jackson ImmunoResearch Laboratories, INC, West Grove, PA, USA). The nuclei were stained by 4′,6-diamidino-2-phenylindole (DAPI). The samples were mounted in aqueous mounting solution (Fluomount-G™, Electron Microscopy Science, Hatfield, PA, USA), and the immunofluorescence images were taken by Olympus epifluorescence microscope (Olympus IX-81, Olympus, Tokyo, Japan).

### Western blot analysis

After treatment, cells in 2D high density culture were rinsed with chilled PBS, and lysed with radioimmunoprecipitation assay buffer (RIPA, ThermoFisher Scientific), containing protease inhibitor and phosphatase inhibit cocktails (ThermoFisher Scientific). The cell lysate was centrifuged at 12000gx at 4 °C and the supernatant was collected and store at − 80 °C. The amount of protein in each sample was quantified by a bicinchoninic acid protein assay kit (Bio-Rad, Hercules, CA, USA) per manufacturer’s instructions. Ten to twenty microgram of protein was resolved by SDS-PAGE, followed by electrophoresed onto polyvinylidene (PVDF) membrane (Pall Corporation, Port Washington, NY, USA). The PVDF membrane was soaked in SuperBlock blocking buffer (ThermoFisher Scientific) at room temperature, followed by incubating with primary antibodies including, anti-SOX9, 1:2000 and anti-collagen II 1:2000 at 4 °C. The membrane was then rinsed with Tris-Buffered Saline-Tween 20 (TBST) and the immunocomplexes were incubated with secondary antibody (1:4000; goat-anti-rabbit) conjugated with horse-raddish peroxidase (code No. 111–035-003, Jackson ImmunoResearch) for SOX9 and collagen II. The immunocomplexes on the PVDF membrane were incubated with the enhanced chemoluminescence reagent (Bio-Rad) and visualized by fluoregraphy on X-ray film (Fujifilm, Tokyo, Japan).

### Screening of aqueous extract of burdock root by high performance liquid chromatography

High performance liquid chromatography (HPLC) was used to screen the chemical constituents in BD root aqueous extract. The HPLC was performed using an Agilent Technologies 1200 series (Agilent, Santa Clara, CA, USA). 10 mg of aqueous extract of burdock root powder was dissolved in 1 ml deionized water and sample was introduced into InertSustain C18 column (250 × 4.6 mm, 5 μm) (GL Sciences, Nakano-ku, Tokyo, Japan). Conditions included a flow rate 1.0 mL/min in elution mode gradient using mixture of acetonitrile and 0.1% acetic acid. Gradient conditions included acetonitrile (25%) and 0.1% acetic acid (75%) at initial 10 min, then acetonitrile (50%) and 0.1% acetic acid (50%) for the next 10 mine, and acetonitrile (25%) and 0.1% acetic acid (75%) for the last 10 mine. The injection volume of the aqueous sample was 10 μL and a run time of 30 min. Chlorogenic acid (1 mg/ml in DMSO, Tocris, Bristol, UK) was used for identification.

### Statistics

The quantified results are reported in mean ± standard error of mean (SEM) based on at least three independent experiments. The statistical analysis was performed by analysis of variance (ANOVA) and Tukey’s Post Hoc Test was carried out to determine significant differences between groups. Significance was accepted when *p* < 0.05.

## Results

**Fig. 1:**
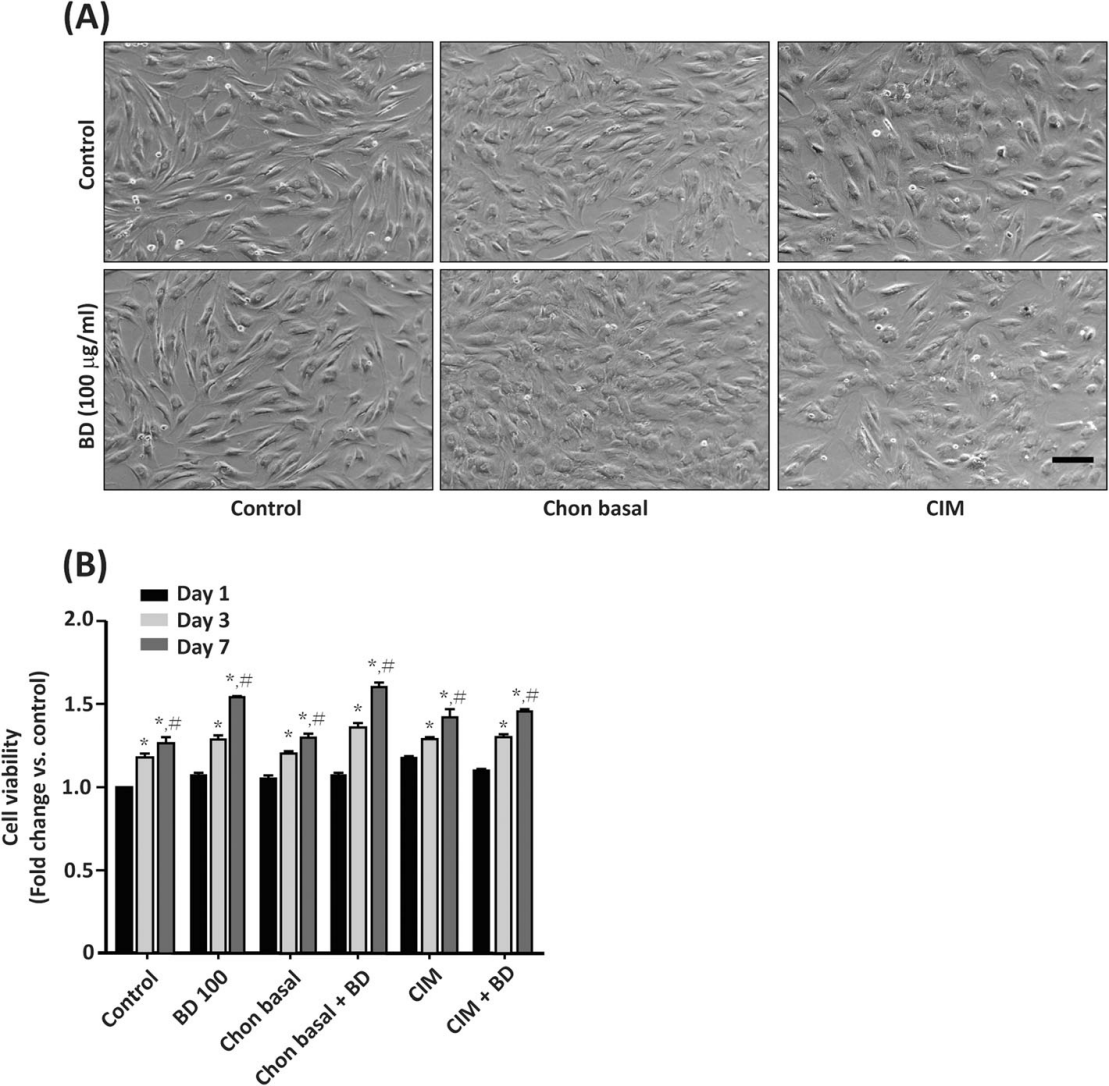
The effects of aqueous extract of *Arctium lappa* L. (Burdock, BD) on mesenchymal stem cell growth. Human bone marrow-derived mesemchymal stem cells (hMSCs) were seeded in a 96-well plate at a density of 2000 cells/well in the presence of control medium for 16 h. Cells were treated with control, BD 100 (100 μg/ml), chon basal, chon basal + BD 100, chondrogenic induction medium (CIM), and CIM + BD 100 for 1, 3, and 7 days. **a** The morphological changes of cells by phase contrast microscopy. **b** Cell viability was measured using MTT assay. The results are presented as mean ± standard error of mean (SEM) of three independent experiments. *, *P* < 0.05 vs. day 1 relevant treatment; #, *P* < 0.05 vs. day 3 relevant treatment. Scale bar: 100 μm

Our first question was if the aqueous extract of burdock root (BD) affected hMSC cell viability. The hMSCs were seeded at a density of 2000 cells/well in a 96-well plate, and treated with serum free medium (Control, containing insulin-transferrin-selenium), BD 100 (Control plus BD 100 μg/ml), chon basal, chon basal + BD (100 μg/ml), CIM, and CIM + BD (100 μg/ml). At day 7, the hMSCs demonstrated elongated fibroblast morphology without treatment (Fig. 1a). In the presence of BD alone, they had elongated morphology with an increased number of cells without evidence of loss (Fig. 1b). After treatment with chon basal or CIM, the morphology of cells became round and shorter than cells without treatment. The morphology after treatment with CIM resembled to that with chon basal. The morphology after treatment with BD combined with chon basal resembled to that with CIM (Fig. 1a). After 1 day of treatment, the cells retained their activity regardless of the treatment, as measured by MTT assay. After 3 days of treatment, we observed a significant increase of cell proliferation in all groups compared with their day 1 relevant treatments. After 7 days, the cell proliferation further increased in all treatments compared with their day 1 and day 3 relevant treatments (Fig. 1b). These results suggest that BD promotes hMSC cell growth without causeing cell death. BD does not change the cell morphology during chondrogenic induction.

**Fig. 2:**
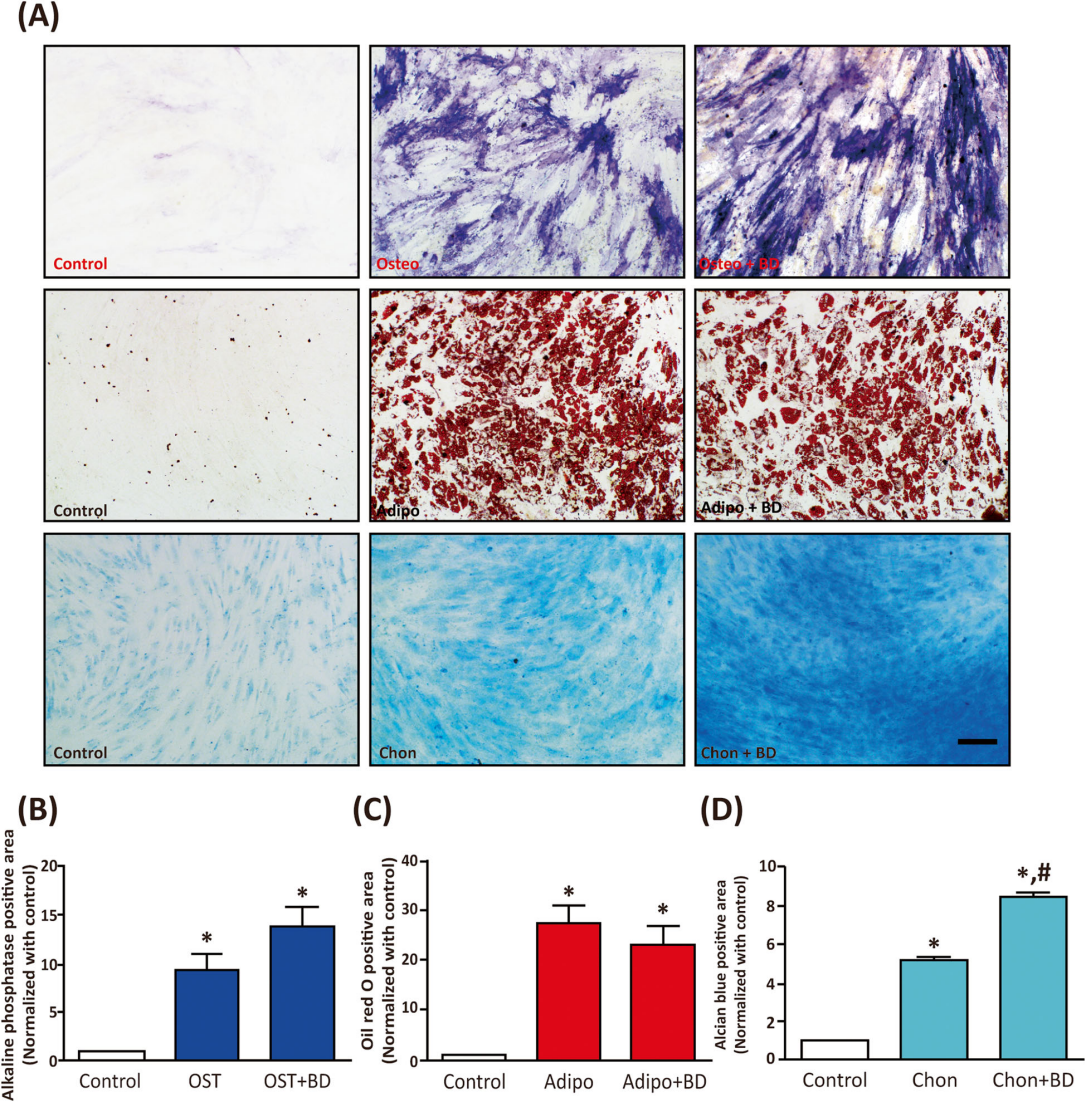
The effects of BD on the differentiation ability of hMSCs. The hMSCs were seeded for 16 h at a density of 3 × 10^3^ cells/cm^2^ for osteogenic induction and at 2 × 10^4^ cells/cm^2^ for adipogenic and chondrogenic induction. Cells were treated with control, OIM, AIM, and CIM in the presence or absence of BD (100 μg/ml) for 14 days. Cells were fixed and stained alkaline phosphatase activity for osteogenic differentiation, see upper panels. Oil red O staining was used for adipogenic differentiation, see middle panels. Alcian blue staining was used for chondrogenic differentiation, see lower panels. Scale bar: 100 μm. **b-d** Quantification results of alkaline phasphatase (**b**), Oil red O (**c**) and (**d**) alcian blue staining was conducted by ImageJ software. The results are presented as mean ± SEM of three independent experiments. *, *P* < 0.05 vs. control; #, *P* < 0.05 vs. Chon

The next question was whether BD altered the differentiation capability of hMSC. To this end, hMSCs were seeded at low density (3000 cells/cm^2^) for osteogenic differentiation, and at high density (20,000 cells/cm^2^) for adipogenic and chondrogenic differentiation [6, 46, 47]. In addition to control, we treated the cells for 14 days with differentiation induction media (OIM, AIM, and CIM) and differentiation induction media plus BD (100 μg/ml). Cell differentiation was examined via staining as per Pittenger et al. [6]. After osteogenic induction, we observed a significant high alkaline phosphatase activity staining in the purple color. The presence of BD slightly increased OIM-induced osteogenic differentiation (Fig. 2a, upper panels, Fig. 2b). AIM treatment significantly increased the accumulation of lipid droplets in cytosol as reflected by oil red O staining, whereas the presence of BD did not alter AIM-induced adipogenic differentiation (Fig. 2a, middle panels, Fig. 2c). Interestingly, CIM treatment increased chondrogenic differentiation as confirmed by the amount of Alcian blue-stained GAGs. The presence of BD significantly enhanced CIM-induced chondrogenic differentiation (Fig. 2a, lower panels, Fig. 2d). These results suggest that BD enhances the chondrogenesis of hMSCs, but does not have effects of enhancing osteogenesis or adipogenesis.

**Fig. 3:**
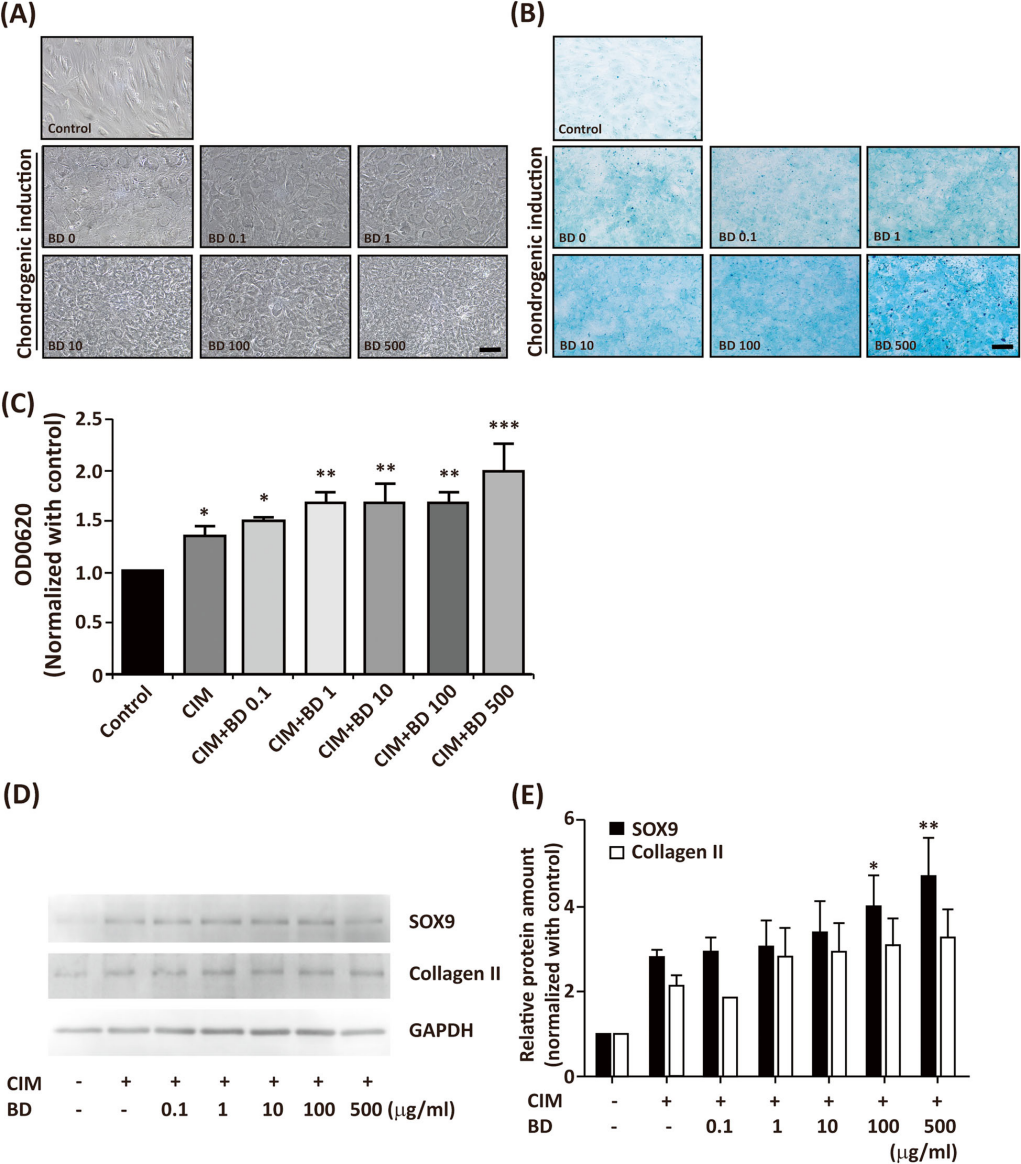
BD enhances CIM-induced chondrogenesis in high density cultures. The hMSCs were seeded at a density of 2 × 10 cells/cm2 for 14 days in the absence or presence of CIM with different dosages of BD (0, 0.1, 1, 10, 100, 500 μg/ml). **a** The morphological changes by phase contract microscopy. **b** The chondrogenic differentiation via Alcian blue staining. **c** The Alcian blue-stained glycosaminoglycans (GAGs) were extracted, and the absorbance was read at OD 620 nm. The results are presented as mean ± SEM from at least 5 independent experiments. *, *P* < 0.05, **, *P* < 0.01, ***, *P* < 0.001 vs. control. Scale bar: 100 μm. **d** hMSCs were seeded at 20000 cells/cm and treated with CIM in the absence or presence of BD (from 0.1-500 μg/ml) for 7 days. Cells were than harvested and the protein levels of SOX-9 and type II collagen were analyzed by Westerm blot. Cell lysate from untreated cells was used as control. **e** Quantification results of (**d**). The results are presented as mean ± SEM of two to three independent experiments. *, *P* < 0.05, **, *P* < 0.01 vs. control

To further confirm whether BD promoted chondrogenesis, we tested the dose dependence of BD in enhancement of CIM-induced chondrogenesis. The hMSCs were seeded at 20000 cells/cm^2^ in the absence or presence of CIM with BD at dosages of 0, 0.1, 1, 10, 100, and 500 μg/ml. The chondrogenic differentiation was assessed by Alcian blue staining. Without treatment, hMSC exhibited a fibroblast-like morphology, as shown in Fig. 3a. After chondrogenic induction, the morphology of hMSC changed from spindle to polygonal or round shape. In the presence of BD, the morphology of BD-treated cells was similar to that of CIM-treated cells. Without chondrogenic induction, the cells produced very few GAGs. After chondrogenic induction, the amount of GAGs increased significantly. In the presence of BD, it dose-dependently enhanced CIM-induced chondrogenic differentiation (Fig. 3b, c). To further characterize whether BD enhanced CIM-induced chondrogenic differentiation, we performed Western blot analysis to examine the protein levels of SOX9 and collagen II. We found that CIM-induced protein levels of SOX9 and collagen II. Co-treatment of BD dose dependently enhanced CIM-induced protein levels of SOX9 and collagen II (Fig. 3d, e; Supplemental Figure 1). The above findings indicate that BD enhances the CIM-induced chondrogenesis-associated marker proteins.

**Fig. 4:**
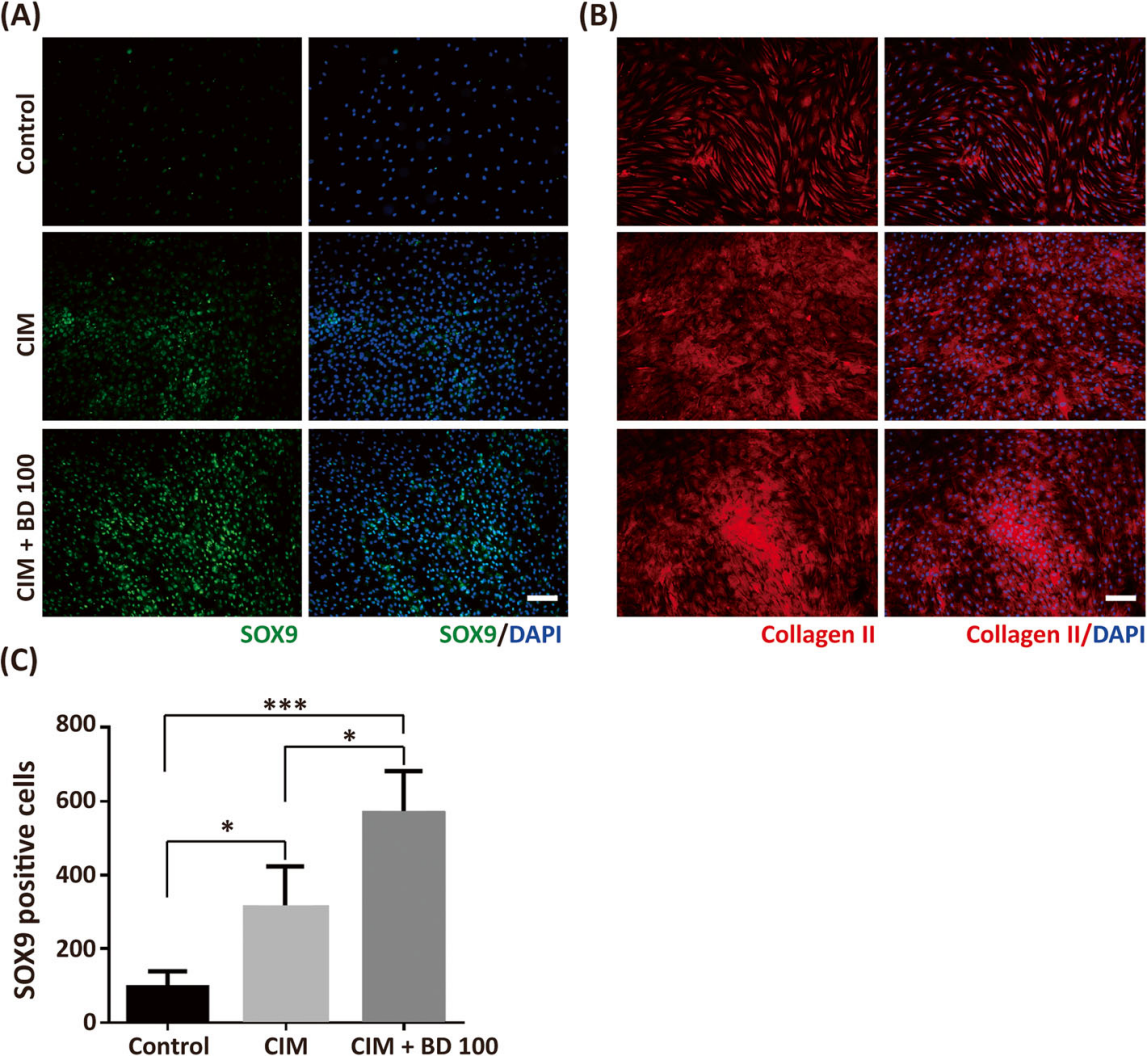
BD enhances CIM-induced chondrogenic markers in high density cultures. The hMSCs were seeded at a density of 2 × 104 cells/cm2 for 7 days in the absence or presence of CIM and CIM plus BD 100 (100 μg/ml). Cells were fixed and stained with (**a**) SOX9 and (**b**) type II collagen (Collagen II). The nuclei were counter stained with DAPI. **c** The SOX9 positively stained cells were counted in the DAPI positive cells. The results are presented as mean ± SEM from four independent experiments. *, *P* < 0.05; ***, *P* < 0.001. Scale bar: 100 μm

To understand the role of BD in the enhancement of chondrogenesis after a 7-day treatment, we performed the immunofluorescence staining of the chondrogenic markers such as nuclear localized chondrogenic transcription factor SOX9 and cartilage matrix collagen II [19, 49]. The control group had very few nuclear localized SOX9 positive cells, as shown in Fig. 4a and c. The treatment of CIM significantly increased the number of nuclear localized SOX9 cells. Co-treatment of BD (100 μg/ml) with CIM further increased the number of CIM-induced nuclear localized SOX9. Similar results were obtained from collagen II staining where only basal amounts of collagen II were found in the control. The CIM treatment increased collagen II staining. Co-treatment of BD promoted CIM-induced collagen II staining (Fig. 4b).

**Fig. 5:**
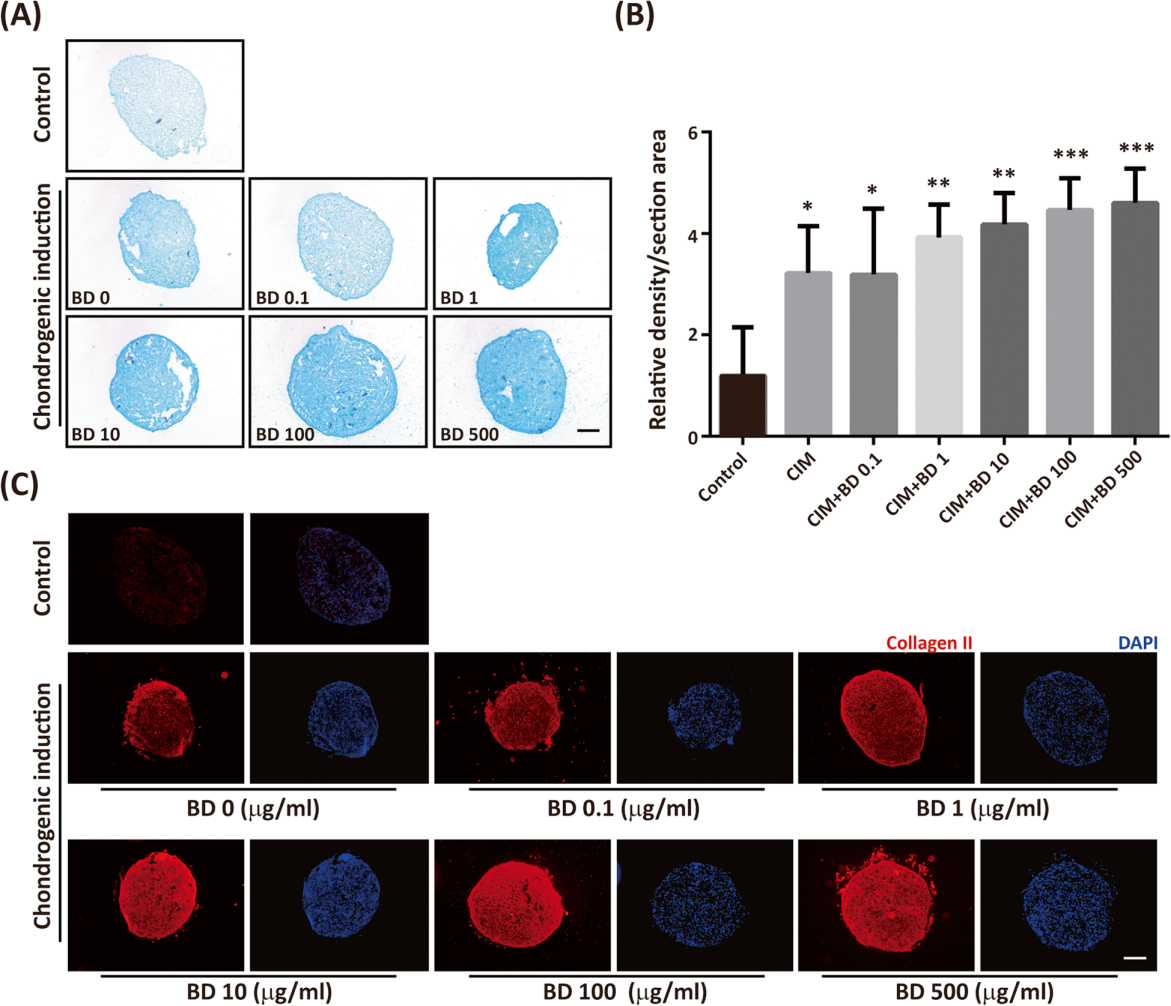
BD enhances CIM-induced chondrogenic differentiation in 3D micromass culture. The 3 × 10^5^ cells were placed into a 15 ml conical tube, and centrifuged to yield micromass. Cells were treated with and without CIM in the absence or presence of BD (0, 0.1, 1, 10, 100, 500 μg/ml) for 14 days. **a** The cell pellet in each tube was cryosectioned and stained with Alcian blue. **b** The Alcian blue stained GAGs were quantified using Image J software. **c** The cryosection pellets were fixed and immunostained with type II collagen (Collagen II). The nuclei were counter stained with DAPI. The results are presented as mean ± SEM from four independent experiments. *, P < 0.05, **, P < 0.01, ***, P < 0.001 vs. control

Though BD together with CIM can promote CIM-induced chondrogenic differentiation in a high density 2-dimensional (2D) culture system, these results may not be representative of chondrogenesis since chondrogenesis usually takes place in a 3-dimensional (3D) environment. To mimic in vivo chondrogenesis, we performed a 3D micromass pellet culture. The micromass pellets were treated for 14 days with and without CIM together with BD of different dosages. The micromass pellets were cryosectioned and stained with Alcian blue after 14 days treatment. The results were analyzed and quantified using ImageJ software. Without CIM treatment, only little Alcian blue-stained GAGs were observed in the control micromass pellets. The amount of GAGs increased significantly with CIM treatment. Co-treatment with BD enhanced the CIM-induced amount of GAGs (Fig. 5a, b). The increment was dependent on the dose of BD. Similar results were obtained by staining cartilage matrix collagen II. The presence of CIM increased the amount of type II collagen significantly. The presence of BD enhanced CIM-induced chondrogenesis (Fig. 5c).

For the induction of chondrogenic differentiation, we performed a chemical-defined chondrogenic induction medium containing chemical supplements and TGF-β [6, 19]. Previous studies demonstrated that TGF-β is a potent chondrogenic inducer regulating chondrogenic events during development and stimulating the chondrogenesis of MSCs [9, 11]. The importance of TGF-β in promoting chondrogenic differentiation is evident from the increasing SOX9 and GAGs deposition, and aggrecan and collagen II synthesis [49].

**Fig. 6:**
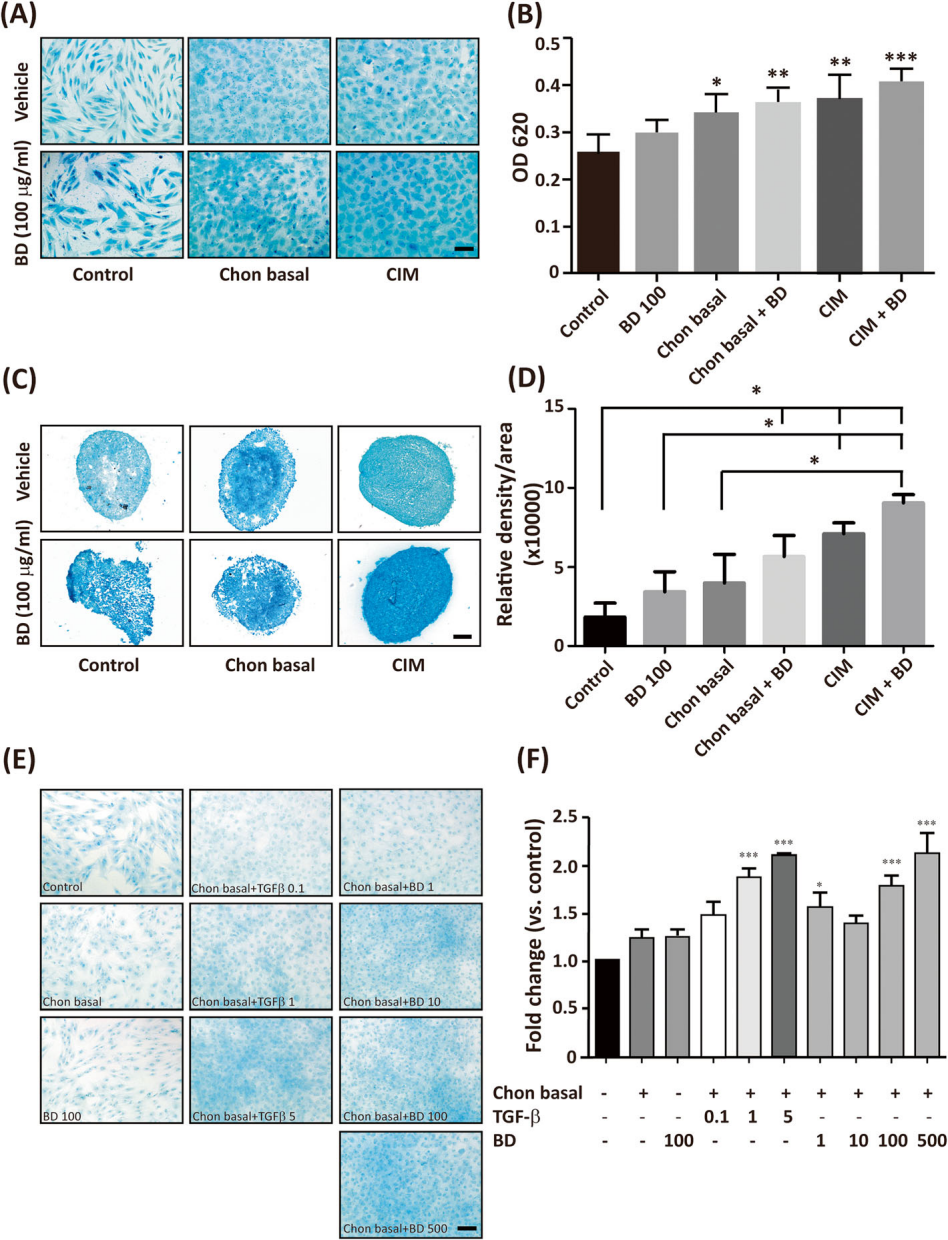
BD treatment is sufficient to induce chondrogenic differentiation. The hMSCs were seeded at a density of 2 × 10^4^ cells/cm^2^ in the absence or presence of chondral basal medium, CIM, and CIM + BD (100 μg/ml) for 14 days. Cells were fixed and stained with Alcian blue. **a** Represented images of chondrogenic differentiated cells. **b** The Alcian blue-stained GAGs were extracted, and their optic density was read at OD 620 nm. The results are presented as mean ± SEM from three independent experiments. *, *P* < 0.05, **, *P* < 0.01, ***, *P* < 0.005 vs. control. **c** The 3 × 10^5^ cells were placed into a 15 ml conical tube, and centrifuged to yield micromass. Cells were treated in the absence or presence of chondral basal medium, CIM, and CIM + BD (100 μg/ml) for 14 days. The cell pellet in each tube was cryosectioned and stained with Alcian blue. **d** The Alcian blue stained GAGs were quantified using Image J software. *, represents *P* < 0.05 vs. related controls, as indicated. Scale bar: 100 μm. **e** hMSCs were seeded at a density of 2 × 10^4^ cells/cm^2^ in the absence or presence of chondral basal medium, BD 100 (100 μg/ml), chon basal plus TGF-β with different doses (from 0.1–5 ng/ml), chon basal plus BD with various doses (from 1 to 500 μg/ml) for 7 days. Cells were fixed and stained with Alcian blue. **f** The Alcian blue-stained GAGs were extracted, and their optic density was read at OD 620 nm. The results are presented as mean ± SEM from three independent experiments. *, *P* < 0.05, **, *P* < 0.01, ***, *P* < 0.001 vs. control. Scale bar: 100 μm

To clarify whether BD alone induces chondrogenic differentiation, we prepared the following chon basal medium: CIM without TGF-β. The hMSCs were seeded at 20000 cells/cm^2^ in the absence and presence of BD (100 μg/ml), chon basal with and without BD (100 μg/ml), or CIM with and without BD (100 μg/ml). The chondrogenic differentiation was accessed by Alcian blue staining. We found a little amount of stained GAGs among the control cells. BD treatment alone slightly increased the staining of GAGs in the control cells. Both chondral basal and CIM significantly increased the amount of GAGs. The presence of BD increased the effect of both chondral basal and CIM (Fig. 6a, b). The treatment with chondral basal medium plus BD induced the same amount of GAGs as CIM did.

Similar results were found using 3D micromass pellets. The presence of BD significantly increased the effect of chondral basal and CIM (Fig. 6c, d). These results implied that BD is equivalent in effect to TGF-β regarding the induction of chondrogenesis. To further examine whether BD treatment alone had equivalent ability to induce chondrogenic differentiation compared with TGF-β, we applied different doses of TGF-β (from 0.1 to 5 ng/ml) and BD (from 1 to 500 μg/ml) in chon basal medium to induce chondrogenic differentiation. Treatment of TGF-β above 1 ng/ml was sufficient to induce chondrogenic differentiation whereas BD at dosage above 100 μg/ml could induce chondrogenic differentiation. Treatment of BD at 100 μg/ml was equivalent to that of TGF-β at 1 ng/ml to induce chondrogenic differentiation whereas BD at dosage of 500 μg/ml was equivalent to that of TGF-β at 5 ng/ml to induce chondrogenic differentiation (Fig. 6e, f). These results suggest that TGF-β may be left out from the chondral basal medium if aqueous extract of BD is added.

**Fig. 7:**
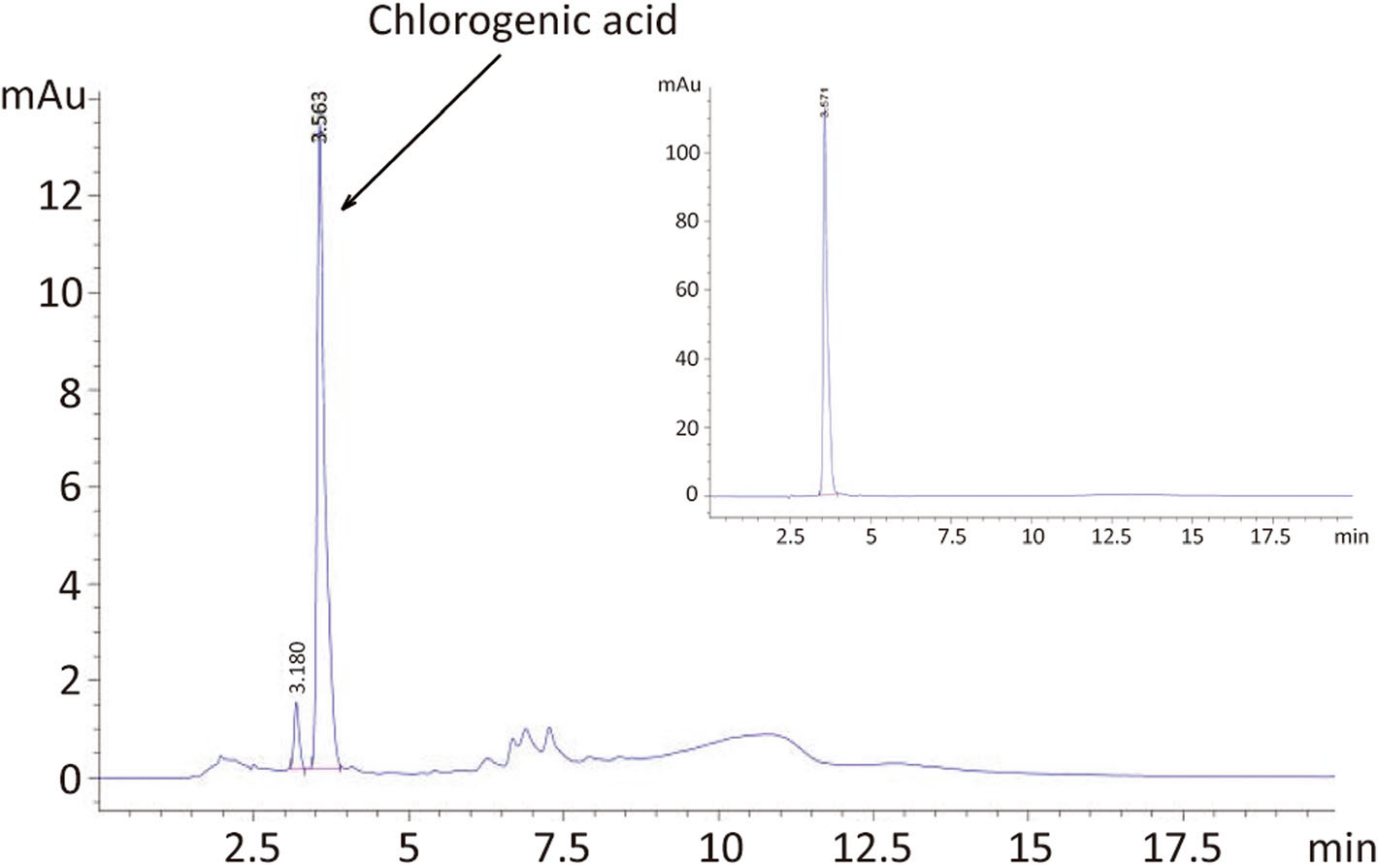
Chromatogram and UV spectrum of the aqueous extract of BD. Aqueous extract of burdock root (10 mg/ml deionized water) was introduced into C18 column and eluted by the gradient using mixture of acetonitrile and 0.1% acetic acid. The representative UV spectrum is shown. (inlet) Chlorogenic acid (1 mg/ml in DMSO) was introduced and the representative UV spectrum is shown

To delineate the fingerprint of aqueous extract of BD, we performed high performance liquid chromatography analysis. Representative chromatogram and UV spectra of aqueous extract of BD determination are presented in Fig. 7. Chromatographic fingerprint analysis of aqueous extract of BD root showed one major peak. By using the purified chemical, we identified this major peak as chlorogenic acid (Fig. 7, inlet).

## Discussion

Our in vitro experiments demonstrated that aqueous extract of burdock root enhances CIM-induced chondrogenic differentiation both in 2D high density and in 3D micromass cultures of hMSCs in the presence of TGF-β. Previous studies performed different formulas to extract burdock roots and the extracts were used for different purposes. For example, Tian et al. applied ethyl acetate extract of burdock roots (EAL) on hydrogen peroxide (H_2_O_2_)-induced cell injury in human SH-SY5Y neuroblastoma cells. They found that EAL served as an antioxidant and possessed a neuroprotective effect [50]. Yang et al. discovered that ethanol extract of BD roots, particularly its active compound oleamide, possessed anti-allergic activity by attenuating the secretion of histamine and allergic cytokines through MAPK-mediated signaling [51]. Other applications of aqueous extract of burdock roots include the attenuation of high fat diet-induced body weight and serum cholesterol [52], the enhancement of male sexual behavior in rats [53], and anti-proliferative and pro-apoptotic effects to leukemia cells [54]. These results suggest that various active ingredients may present in burdock root extracts with different solvents.

In the current study, we demonstrated that treatment with aqueous extract of burdock root did not induce cytotoxic effect to hMSCs. Indeed, it increased cell proliferation after 7 days of treatment in a serum free medium at the dosage of 100 μg/ml (Fig. 1b). We also discovered that aqueous extract of burdock root enhanced CIM-induced chondrogenesis, but with little to no effect on enhancing adipogenesis and osteogenesis. SOX9 is a transcription factor required for chondrogenesis [18]. The activation of SOX9 regulates its downstream proteins such as collagen II [49, 55–57], cartilage oligomeric matrix protein [56, 57], and aggrecan [58]. We demonstrated that the aqueous extract of burdock roots enhanced TGF-β based CIM-induced chondrogenic differentiation through increasing the protein levels and nuclear localization of SOX9. We also found enhanced expression of SOX9 downstream chondrogenic matrix proteins such as collagen II. The production of chondrogenic specific proteoglycans such as chondrogenic specific GAGs also increased.

TGF-β is known as a pleiotropic cytokine involved in the regulation of cell proliferation, apoptosis, the suppression of the immune system, and the regulation of development [59–61]. TGF-β is also an essential cytokine for chondrogenic differentiation through the activation of its downstream effect SMAD [62–64]. Previous studies and our experiments demonstrated that TGF-β-containing CIM successfully induced chondrogenic differentiation of hMSCs in 2D and 3D culture systems [6, 19, 47]. We found that aqueous extract of burdock root enhanced CIM-induced chondrogenic differentiation. The aqueous extract of burdock roots may act through potentiating TGF-β downstream signaling. The effective dosage of active component(s) in the aqueous extract of burdock root for enhancing CIM-induced chondrogenesis was higher than 10 μg/ml for both 2D and 3D micromass cultures, which was not very high. With burdock, we found an equivalent chondrogenic induction capability to that of TGF-β in CIM. However, whether BD treatment-induced chondrogenic differentiation is through the induction of TGF-β remains to be elucidates. Previous studies reported that other than TGF-β, several growth factors such as FGFs, BMPs, and IGF-1, are reported to induce chondrogenesis both in vitro and in vivo [9–12, 25]. These findings suggest that the above growth factors may mediate BD-induced chondrogenic differentiation. Together, this aqueous extract of burdock root slightly increased cell proliferation but not chondrogenic differentiation. This suggests the need of supplemental materials in the chondral basal medium to induce chondrogenesis in the presence of BD.

On the other hand, our chromatogram and UV spectrum study showed a major chemical, the chlorogenic acid in aqueous extract of BD. Previous findings indicated several major compounds in the BD root extract, such as arctiin and arctigenin [43]. Arctiin and arctigenin belong to lignans family and are found to possess numerous effects on the treatment of different diseases [43]. These chemicals are mainly found in the extraction of organic solvent [29] due to their structural nature. Therefore, we anticipated that our aqueous extract of BD may not contain these chemicals. Interestingly, the major peak in our chromatogram was found to be chlorogenic acid. Cheng et al., have discovered that applying chlorogenic acid in an alginate scaffold improved repair of damaged articular cartilage [65]. This study implies that BD-induced chondrogenic differentiation may be mediated by chlorogenic acid. The detail molecular mechanisms underlying BD-induced chondrogenic differentiation remains to be elucidated.

Maghsoumi-Norouzabad et al. reported the use of burdock root tea for the treatment of knee OA. They gave three cups of burdock root tea (2 g/150 ml water) daily for 42 days to their patients. As a result, the amount of inflammatory markers decreased, while the anti-oxidative capability, such as both glutathione peroxidase and superoxide dismutase activities were increased significantly [33]. Other than aqueous extract of burdock root, Cai et al. reported that four traditional Chinese herbs promoted the proliferation and the chondrogenesis of bone marrow-derived mesenchymal stem cells [66]. We anticipate that the aqueous extract of burdock root can be used as an alternative strategy for the treatment of musculoskeletal diseases.

## Conclusion

Taken together, our finding suggests that the aqueous extract of burdock root can promote CIM-induced chondrogenic differentiation or this aqueous extract of burdock root can be used alone to stimulate chondrogenic differentiation. The above study indicates that the aqueous extract of burdock root can be used as alternative strategy in the induction of chondrogenesis for treatment purpose. Further studies to identify the active ingredients in the aqueous extract of burdock root will be elucidated.

## Supplementary Information

**Additional file 1:** **Figure S1.** The un-cropped Western blot results of Fig. 3d.

## Data Availability

The datasets used and/or analyzed during the current study are available from the corresponding author on reasonable request.

## Abbreviations

OA: Osteoarthritis
CHM: Chinese herbal medicine
TCM: Traditional Chinese medicine
hMSC(s): Human mesenchymal stem cell(s)
DMEM: DULBECCO’S Modified Eagles Medium
TGFβ: Transforming growth factor β
FGF: Fibroblast growth factor
BMP: Bone morphogenic protein
IGF-1: Insulin-like growth factor-1
SOX9: Sry-box transcription factor 9
GAG(s): Glycosaminoglycan(s)
BD: Aqueous extract of burdock root
BM: Basal medium
Chon basal: Chondrogenic induction medium without TGFβ
CIM: Chondrogenic induction medium
OIM: Osteogenic induction medium
AIM: Adipogenic induction medium
MTT: 3-(4, 5 Dimethylthiazol-2-yl)-2,5-Diphenyltetrazolium Bromide
2D: 2 dimensional
3D: 3 dimensional
EAL: Ethyl acetate extract of burdock roots
H_2_O_2_: Hydrogen peroxide
