# Supplementary figures and images for: Aqueous extract of Arctium lappa L. root (burdock) enhances chondrogenesis in human bone marrow-derived mesenchymal stem cells
Source: BMC Complement Med Ther. 2020 Nov 23;20:364. doi: 10.1186/s12906-020-03158-1 (PMC7686739; doi:10.1186/s12906-020-03158-1)

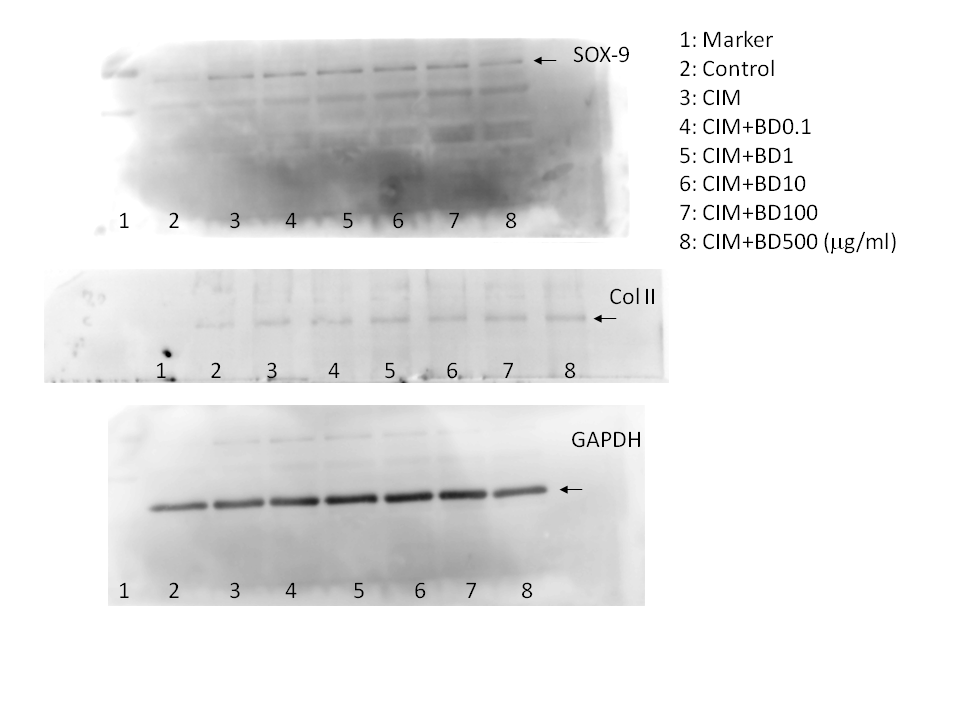

Supplement: Supplementary file 1 — Additional file 1: Figure S1. The un-cropped Western blot results of Fig. 3d. [file 12906_2020_3158_MOESM1_ESM.tif]
